# Supplementary material for: Unique phenotypes and clonal expansions of human CD4 effector memory T cells re-expressing CD45RA
Source: Nat Commun. 2017 Nov 13;8:1473. doi: 10.1038/s41467-017-01728-5 (PMC5684192; doi:10.1038/s41467-017-01728-5)
Supplement: Supplementary file 1 — Supplementary Information [file 41467_2017_1728_MOESM1_ESM.pdf]

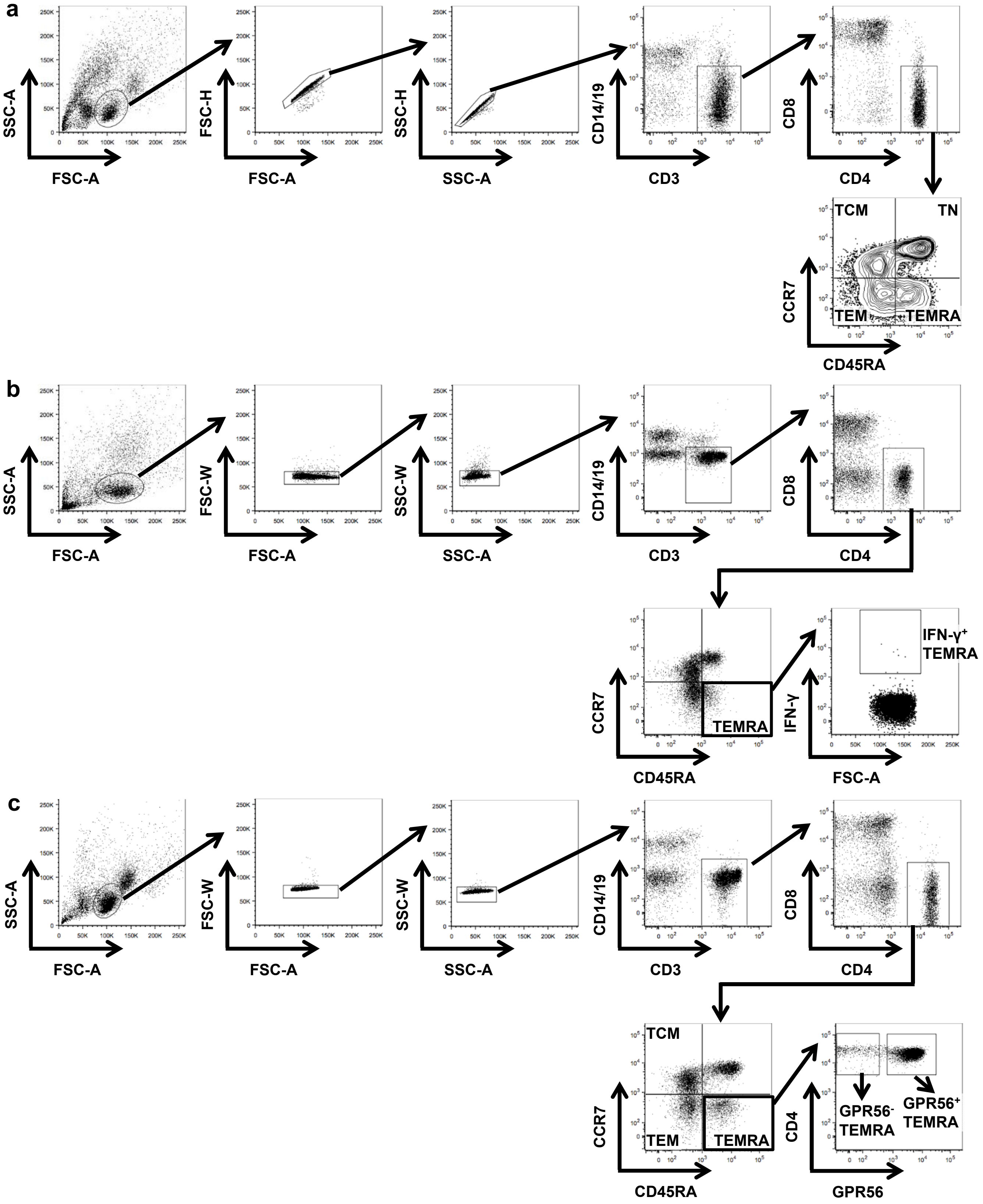

Supplementary Figure 1. Gating strategies. (**a**) Gating strategy to identify and sort CD4 T cell subsets corresponding to Fig. 1a. (**b**) Gating strategy to identify CD4 effector memory T cells re-expressing CD45RA (TEMRA) and IFN-γ^+ CD4 TEMRA cells corresponding to Fig. 4a, 7a, 7b, 8a, 8b. (**c**) Gating strategy to sort TCM, TEM, GPR56$^-$ TEMRA and GPR56$^+$ TEMRA cells for TCR sequencing presented in Fig. 6b-d. The same strategy was used to sort GPR56$^-$ and GPR56$^+$ TEMRA cells for *in vitro* stimulation presented in Fig. 7d.

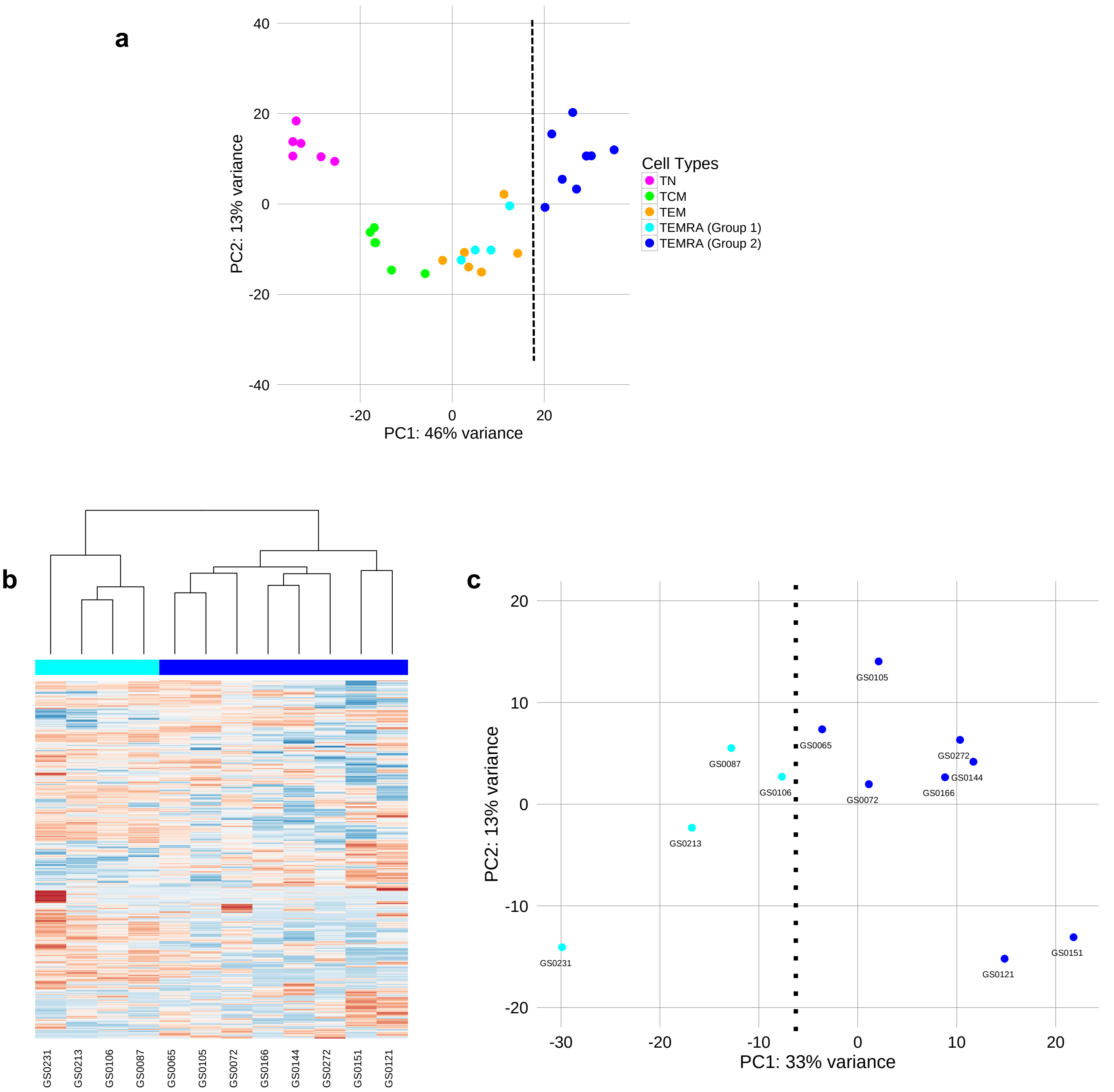

Supplementary Figure 2. CD4 effector memory T cells re-expressing CD45RA (TEMRA) display heterogeneous gene expression profiles between donors. (**a**) PCA analysis of gene expression data (top 500 variable genes) for different CD4 T cell subsets (n = 6 for naïve (TN), central memory (TCM), and effector memory (TEM), and n = 12 for TEMRA). The dashed vertical line separates TEMRA cells from group 2 donors from all other cell subsets. (**b**) Clustering of gene expression data using heatmap (n = 12). (**c**) PCA analysis identified CD4 TEMRA cells from group 1 and group 2 donors (n = 12). In (**b**) and (**c**) only the most variable genes (SD>1) were considered.

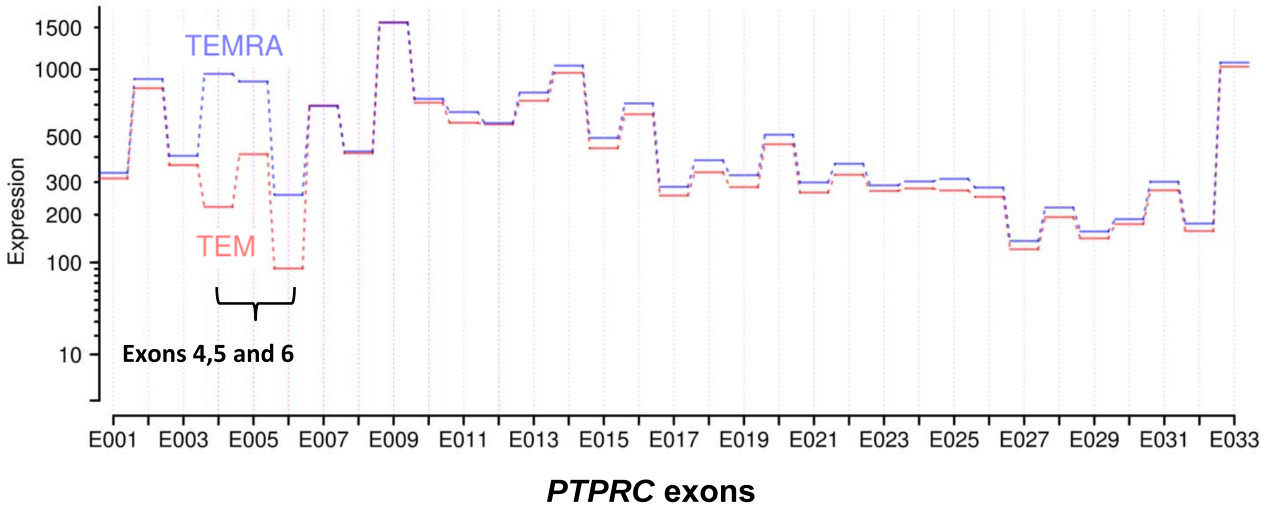

Supplementary Figure 3. The expression of *PTPRC* (encodes CD45) exons 4, 5, and 6 is higher in CD4 effector memory T cells re-expressing CD45RA (TEMRA) than effector memory T (TEM) cells. Plot shows the expression of *PTPRC* exons by CD4 TEMRA (blue) and TEM (red) cells (n = 6).

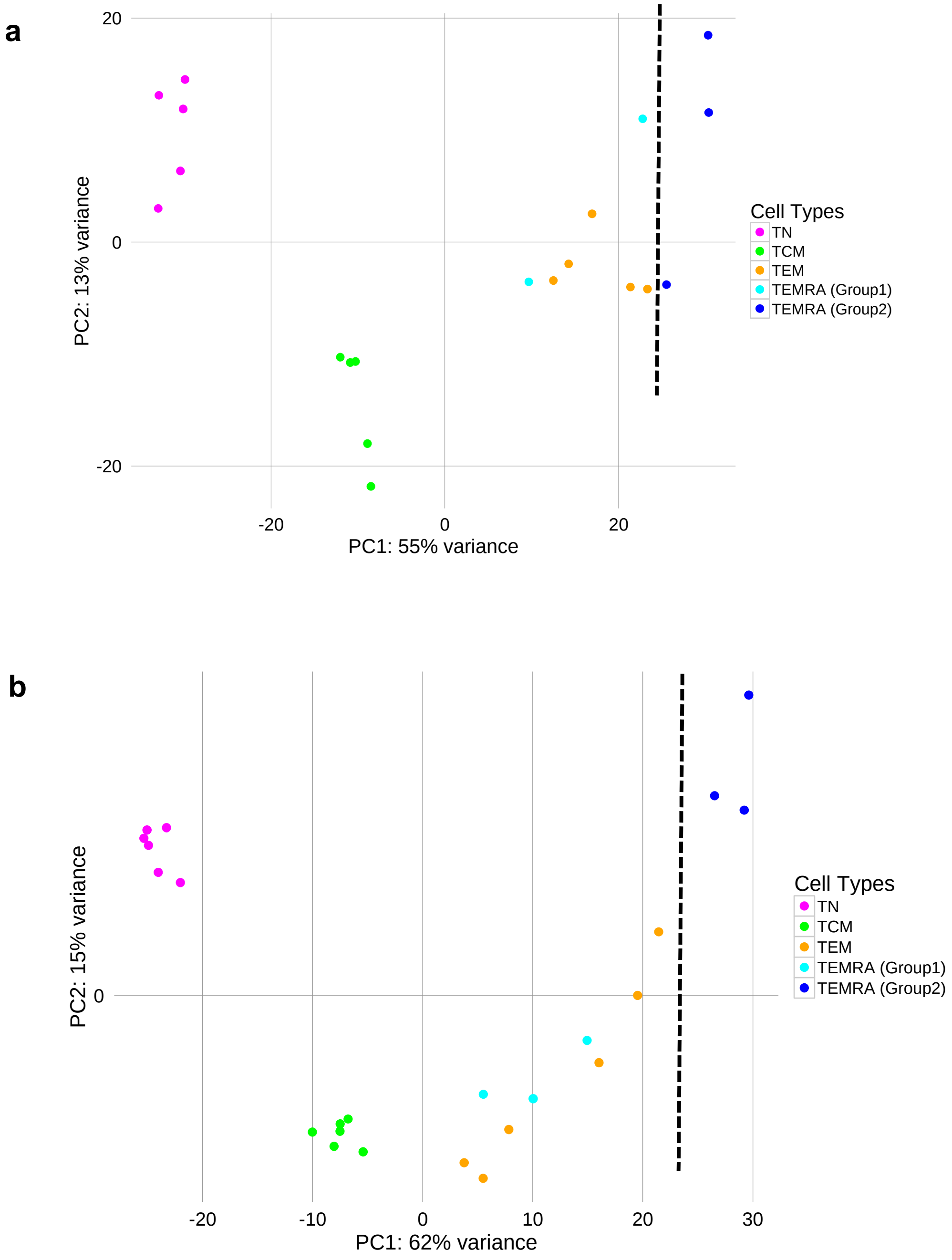

Supplementary Figure 4. The identification of group 1 and group 2 donors in additional cohorts. PCA analysis of gene expression data (using the top 1000 variable genes from Fig. 1b) for the different CD4 T cell types from (**a**) DENV seropositive donors from Sri Lanka (cohort 2, n = 5) and **(b)** DENV seronegative donors from San Diego (cohort 3, n = 6). Note that the dashed vertical line separates CD4 effector memory T cells re-expressing CD45RA (TEMRA) from group 2 donors from all other cell types.

a

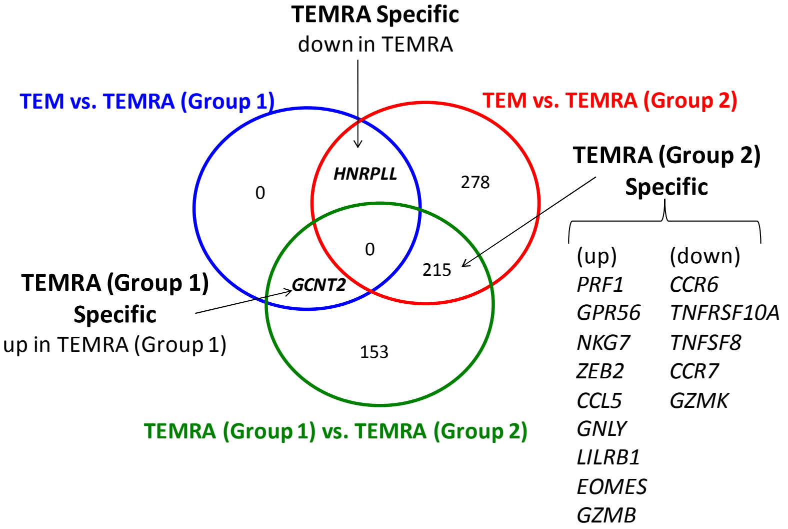

b

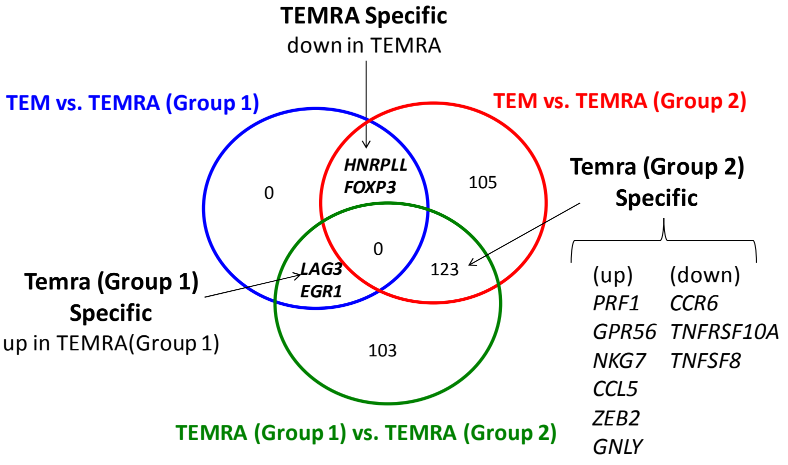

Supplementary Figure 5. Identification of specific transcriptomic profiles for CD4 effector memory T cells re-expressing CD45RA (TEMRA) from group 1 and group 2 donors. Pairwise differential expression analysis between CD4 effector memory T (TEM) cells, CD4 TEMRA cells from group 1 donors, and CD4 TEMRA cells from group 2 donors for (**a**) cohort 1 (n = 6, 4 and 8 for TEM, TEMRA cells from group 1 donors and TEMRA cells from group 2 donors, respectively) and (**b**) cohort 2 and 3 (n = 11, 5 and 6 for TEM, TEMRA cells from group 1 donors and TEMRA cells from group 2 donors, respectively).

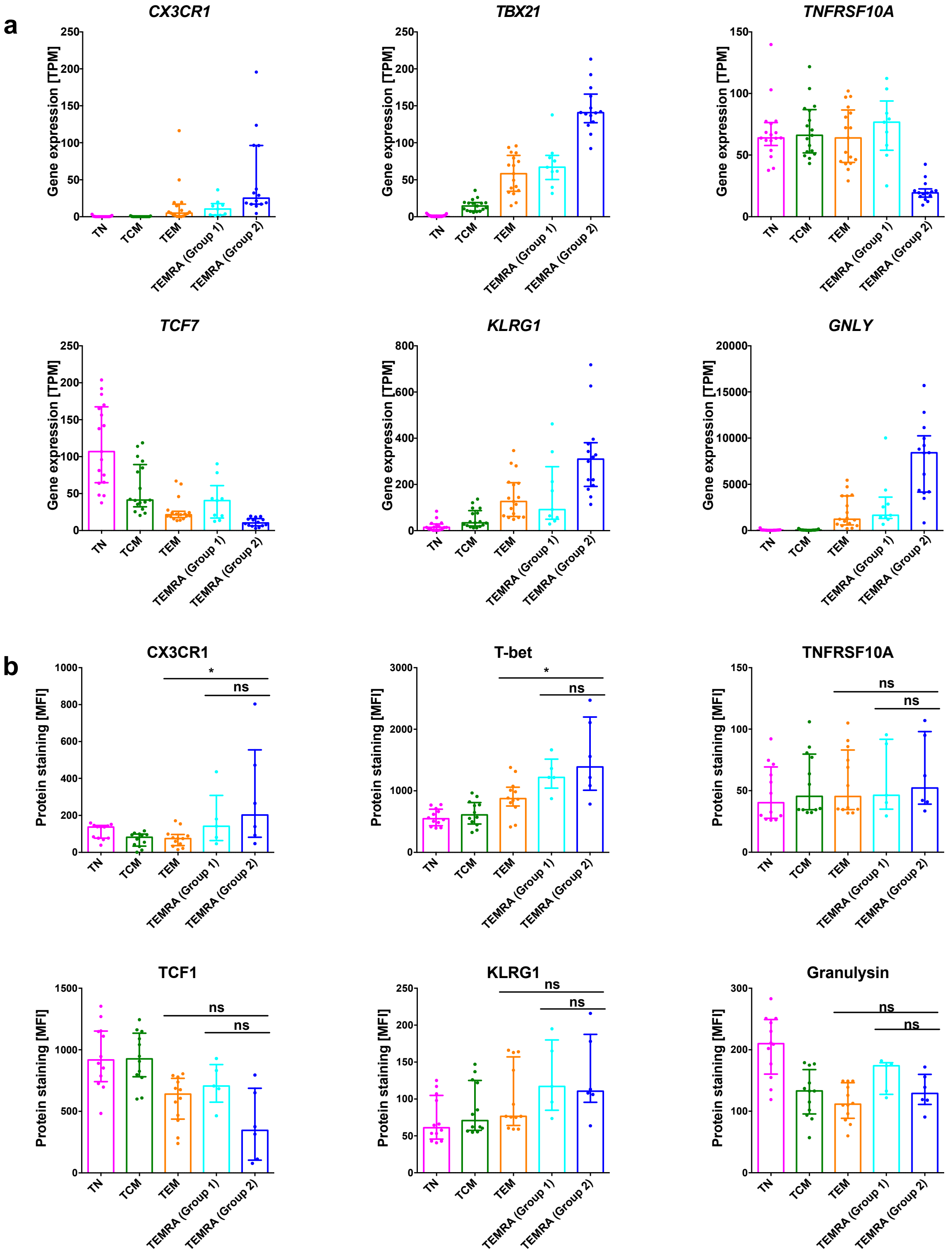

Supplementary Figure 6. Molecules that are differentially expressed by CD4 effector memory T cells re-expressing CD45RA (TEMRA) from group 2 donors at the mRNA-level, but not always at the protein level. (**a**) Bar graphs show gene expression values in transcripts per million (TPM) (n = 17 for naïve (TN), central memory (TCM) and effector memory (TEM), and n = 9 and 14 for TEMRA cells from group 1 and group 2 donors, respectively). (**b**) Bar graphs show protein abundance in mean fluorescence intensity (MFI) (n = 11 for TN, TCM and TEM, and n = 5 and 6 for TEMRA cells from group 1 and group 2 donors, respectively). Error bars show median with interquartile range. Statistical significance was determined by two-tailed Mann-Whitney test. *$p < 0.05$.

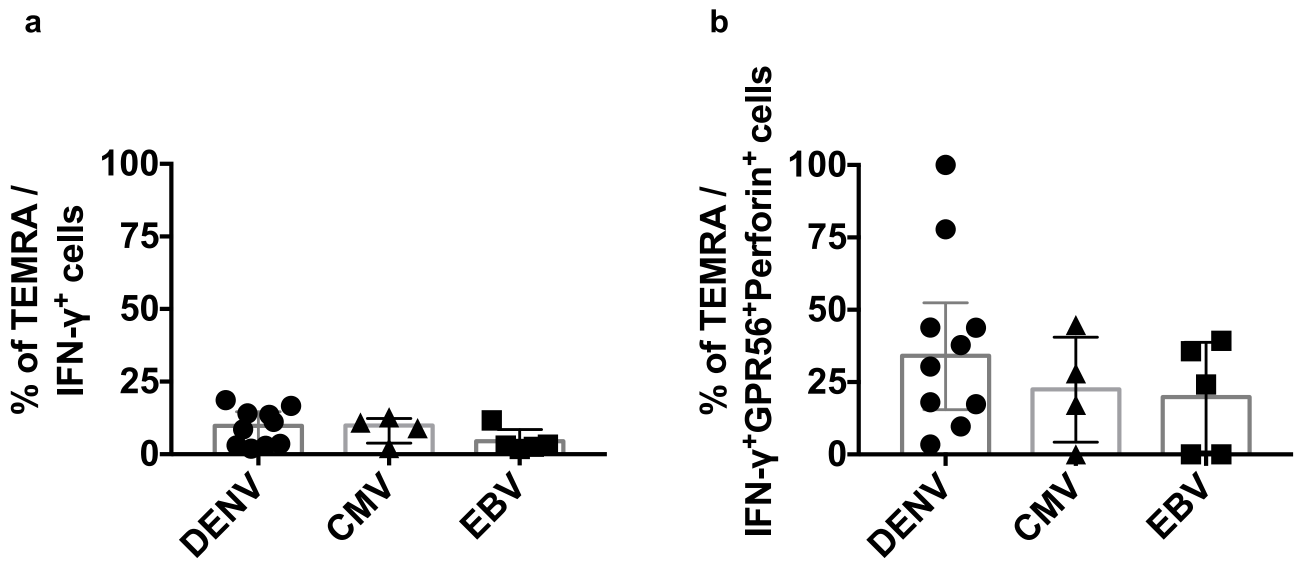

Supplementary Figure 7. CD4 effector memory T cells re-expressing CD45RA (TEMRA) are associated with a specific set of biological phenotypes. Bar graphs show the percentages of TEMRA cells among (**a**) IFN-γ^+ and (**b**) IFN-γ^+GPR56$^+$Perforin$^+$ CD4 T cells (n =10, 4, and 5 for dengue virus (DENV), cytomegalovirus (CMV) and pstein-Barr virus (EBV), respectively). Error bars show median with interquartile range.

Cohort	Donor Code	Region	Gender	Age	DENV Serological Status	CMV Serological Status	EBV Serological Status	Cell Types Contributed	% of Temra/CD4	% of GPR56^{+}Perforin^{+} Temra/CD4	% of GPR56^{-}Perforin^{-} Temra/CD4
1	GS0106	Colombo, Sri Lanka	N/A	N/A	Negative	Positive	Positive	TEMRA	1.1	N/A	N/A
1	GS0213	Colombo, Sri Lanka	N/A	N/A	Negative	Positive	Positive	TEMRA	4.7	N/A	N/A
1	GS0231	Colombo, Sri Lanka	N/A	N/A	Secondary	Positive	Positive	All	7.0	N/A	N/A
1	GS0087	Colombo, Sri Lanka	N/A	N/A	Secondary	Positive	Positive	All	5.0	N/A	N/A
1	GS0166	Colombo, Sri Lanka	N/A	N/A	Negative	Positive	Positive	TEMRA	2.4	N/A	N/A
1	GS0105	Colombo, Sri Lanka	N/A	N/A	Secondary	Positive	Negative	All	15.8	N/A	N/A
1	GS0272	Colombo, Sri Lanka	N/A	N/A	Secondary	Positive	Positive	All	7.0	N/A	N/A
1	GS0065	Colombo, Sri Lanka	N/A	N/A	Secondary	Positive	Positive	All	10.0	N/A	N/A
1	GS0144	Colombo, Sri Lanka	N/A	N/A	Secondary	Positive	Positive	All	8.0	N/A	N/A
1	GS0072	Colombo, Sri Lanka	N/A	N/A	Secondary	N/A	N/A	TEMRA	2.0	N/A	N/A
1	GS0121	Colombo, Sri Lanka	N/A	N/A	Secondary	Positive	Positive	TEMRA	2.2	N/A	N/A
1	GS0151	Colombo, Sri Lanka	N/A	N/A	Secondary	Positive	Positive	TEMRA	16.2	N/A	N/A
2	GS0089	Colombo, Sri Lanka	N/A	N/A	Secondary	Positive	Positive	All	0.6	0.1	0.4
2	GS0098	Colombo, Sri Lanka	N/A	N/A	Secondary	Positive	Positive	All	0.4	0.1	0.1
2	GS0108	Colombo, Sri Lanka	N/A	N/A	Secondary	Positive	Positive	All	2.6	1.7	0.6
2	GS0117	Colombo, Sri Lanka	N/A	N/A	Secondary	Positive	Positive	All	6.4	5.0	0.8
2	GS0259	Colombo, Sri Lanka	N/A	N/A	Secondary	Positive	Positive	All	18.0	17.2	0.3
3	TU0079	San Diego, USA	Male	31	Negative	N/A	N/A	All	0.5	0.1	0.3
3	TU0084	San Diego, USA	Male	24	Negative	N/A	N/A	All	0.4	0.0	0.3
3	TU0085	San Diego, USA	Male	26	Negative	N/A	N/A	All	0.8	0.1	0.6
3	TU0053	San Diego, USA	Male	51	Negative	N/A	N/A	All	17.1	13.7	1.4
3	TU0054	San Diego, USA	Male	60	Negative	N/A	N/A	All	6.2	4.5	1.0
3	TU0057	San Diego, USA	Male	44	Negative	N/A	N/A	All	8.3	6.8	0.6

Supplementary Table 1. Donor Information.

Target	Fluorochrome	Clone	Vendor	Dilution
CCR6	Brilliant Violet 650	G034E3	BioLegend	1/50
CCR7	PerCP Cy5.5	G043H7	BioLegend	1/25
CD3	Alexa Fluor 700	OKT3	BioLegend	1/100
CD4	APC eFluor 780	RPA-T4	eBioscience	1/100
CD8	Brilliant Violet 650	RPA-T8	BioLegend	1/50
CD14	V500	M5E2	BD Biosciences	1/50
CD19	V500	HIB19	BD Biosciences	1/50
CD45RA	eFluor 450	HI100	eBioscience	1/25
CD244	APC	eBioDM244	eBioscience	1/50
CX3CR1	PE Cy7	2A9-1	BioLegend	1/100
GPR56	PE Cy7	CG4	BioLegend	1/50
Granzyme B	PE	GB11	eBioscience	1/20
Granulysin	Alexa Fluor 647	DH2	BioLegend	1/20
Hobit	Alexa Fluor 647	Sanquin-Hobit/1	BD Biosciences	1/20
IFN-γ	PE	4S.B3	eBioscience	1/20
KLRG1	FITC	2F1	BioLegend	1/50
Perforin	FITC	dG9	eBioscience	1/20
Runx3	PE	R3-5G4	BD Biosciences	1/20
T-bet	PE Cy7	eBio4B10	eBioscience	1/20
TCF1	PE	S33-966	BD Biosciences	1/20
ThPOK	PE	6/hcKrox	BD Biosciences	1/20
TNFRSF10A	PE	DJR1	BioLegend	1/50

Supplementary Table 2. Antibodies used in flow cytometry analysis.

Target	Metal	Clone	Vendor
CD3*	115In	UCHT1	Biolegend
CD4	145Nd	RPA-T4	Fluidigim
CD8a	146Nd	RPA-T8	Fluidigim
CD19	142Nd	HIB19	Fluidigim
CD14*	142Nd	M5E2	Biolegend
CD45RA	143Nd	HI100	Fluidigim
CCR6 (CD196)	141Pr	G034E3	Fluidigim
CCR7 (CD197)	159Tb	G043H7	Fluidigim
CX3CR1	172Yb	2A9-1	Fluidigim
GPR56*	158Gd	GG4	Biolegend
CD45RO	149Sm	UCHL1	Fluidigim
CD11b (Mac-1)	209Bi	ICRF44	Fluidigim
S1P5/EDG-8*	151Eu	282503	R&D Systems
CD355 (CRTAM)*	169Tm	Cr24.1	Biolegend
CD85j (ILT2)	156Gd	GHI/75	Fluidigim
CD261*	147Sm	DJR1	Biolegend
S1P1/EDG-1*	163Dy	218713	R&D Systems
CD103*	153Eu	Ber-ACT8	Biolegend
KLRG1*	152Sm	13F12F2	eBioscience
CD38	167Er	HIT2	Fluidigim
CD69	162Dy	FN50	Fluidigim
CD244 (2B4)*	168Er	C1.7	Biolegend
CD278/ICOS	148Nd	C398.4A	Fluidigm
CD152 (CTLA-4)	170Er	14D3	Fluidigm
HLA-DR	174Yb	L243	Fluidigim
Granulysin	176Yb	DH2	Biolegend
IFN-γ	165Ho	B27	Fluidigim
Granzyme B	173Yb	GB11	Fluidigim
Perforin	175Lu	B-D48	Fluidigim
CD107a (LAMP1) *	160Gd	H4A3	Biolegend
Hobit (ZNF683) *	164Dy	Sanquin-Hobit/1	BD Biosciences
TCF-7/TCF-1*	166Er	7F11A10	Biolegend
Tbet	161Dy	4B10	Fluidigim
EOMES*	154Sm	WD1928	eBioscience
Blimp1*	171Yb	ROS195G	Biolegend
KLF2*	155Gd	665333	R&D Systems
ThPOK*	144Nd	11H11A14	Biolegend
RUNX3*	150Nd	R3-5G4	BD Biosciences

*: Antibodies were conjugated in house using the Maxpar Antibody Labeling Kit (Fluidigm) according to the manufacturer's instructions.

Supplementary Table 3. Antibodies used in mass cytometry analysis.

Gene	Expr Log Ratio	Predicted effect
MYC	-1.231	↓
CD28	-1.435	↓
NET1	-1.319	↓
LGALS9	-1.157	↓
CES1	1.261	↓
KLRK1	1.294	↓
ADRB2	1.059	↓
PLCL1	-1.551	↓
MATK	1.322	↓
CCR6	-1.992	↓
LGALS3	-1.145	↓
CAPG	-1.346	↓
CCR4	-1.281	↓
VIPR1	-1.65	↓
TNFSF8	-1.296	↓
FASN	-1.08	↓
GPR183	-1.235	↓
AQP3	-2.163	↓
RGS3	1.241	↓
LPAR2	-1.175	↓
MGAT5	-1.372	↓
MAP3K1	-1.516	↓
FUT7	-1.719	↓
IL6R	-1.129	↓
INADL	-1.239	↓
IRS2	-1.702	↓
CCR10	-1.711	↓
TIAM1	-2.796	↓
TNFRSF25	-1.045	↓
WNT7A	-1.407	↓
FHL1	-1.419	↓
LGALS3BP	-1.125	↓
CYSLTR1	-1.172	↓
LTB	-1.899	↓
PRKCA	-1.219	↓
CAMK4	-1.377	↓
PRF1	1.673	↓
EFNA5	1.631	↓
LEF1	-1.206	↓
IL23A	-1.263	↓
TXK	-1.767	↓
PELI1	-1.079	↓
PDE4DIP	-1.27	↓
GPR56	1.713	↓
CCDC141	-1.191	↓
PTPN12	1.194	↑
GZMB	1.643	↑
FZD4	1.601	↑
ERBB2	1.643	↑
S1PR5	1.255	↑
ADAM19	-1.808	↑
CCL5	1.378	↑
TBX21	1.029	↑
TRAF3IP2	-1.084	↑
DPP4	-1.337	↑
MLLT4	-1.15	↑
NT5E	-1.314	↑
ZEB2	1.286	↑
CX3CR1	1.408	↑
NCF4	-1.473	↑
ADAM12	-1.497	↑
FOXP3	-1.811	↑
PROK2	1.324	↑
GNLY	1.306	↑
IL2RA	-1.272	↑
AIRE	-1.251	↑
TNFRSF10A	-1.604	↑
F2R	1.374	↑
FGR	1.294	↑
LGR6	1.328	↑
SPON2	1.356	↑
CMTM8	-1.297	↑
B3GAT1	1.372	↑
AMICA1	-1.126	Unknown
LY96	-1.304	Unknown
LYPD3	-1.389	Unknown
RORC	-1.543	Unknown
ABI3	1.211	Unknown

Supplementary Table 4. Genes involved in migration.

Gene	Expr Log Ratio	Predicted effect
KLRK1	1.294	↑
GZMB	1.643	↑
LGALS3	-1.145	↑
CCL5	1.378	↑
IFNGR2	-1.543	↑
TBX21	1.029	↑
CD300A	1.114	↑
FOXP3	-1.811	↑
PRF1	1.673	↑
CD244	1.412	↑
FGR	1.294	↑
SLAMF7	1.46	↑
CD28	-1.435	↓
TOP2A	-1.435	↓
ERBB2	1.643	↓
KLRD1	1.785	↓
IL23A	-1.263	↓
GPR56	1.713	Unknown
CD27	-1.527	Unknown
PRKCA	-1.219	Unknown

Supplementary Table 5. Genes involved in cytotoxicity**.**

Cell type / Donor	TN	TCM	TEM	TEMRA (Group 1)	TEMRA (Group 2)
**GS0065**	0.03	0.03	0.05	NA	0.37
**GS0072**	NA	NA	NA	NA	0.39
**GS0121**	NA	NA	NA	NA	0.62
**GS0151**	NA	NA	NA	NA	0.25
**GS0106**	NA	NA	NA	0.19	NA
**GS0166**	NA	NA	NA	NA	0.72
**GS0213**	NA	NA	NA	0.03	NA
**GS0272**	0.03	0.02	0.23	NA	0.54
**GS0105**	0.02	0.03	0.03	NA	0.31
**GS0231**	0.03	0.01	0.03	0.04	NA
**GS0087**	0.03	0.02	0.02	0.09	NA
**GS00144**	0.02	0.02	0.06	NA	0.38
**GS0079**	0.01	0.01	0.04	0.09	NA
**GS0098**	0.01	0.01	0.01	0.01	NA
**GS0108**	0.01	0.01	0.05	NA	0.13
**GS0117**	0.01	0.01	0.04	NA	0.17
**GS0259**	0.01	0.01	0.04	NA	0.25
**TU0053**	0.01	0.02	0.06	0.13	NA
**TU0054**	0.01	0.01	0.18	NA	0.41
**TU0057**	0.01	0.01	0.01	0.03	NA
**TU0079**	0.01	0.01	0.08	0.15	NA
**TU0084**	0.01	0.01	0.25	NA	0.39
**TU0085**	0.01	0.01	0.08	NA	0.18
**Median**	0.01	0.01	0.05	0.09	0.375

Supplementary Table 6. Comparison of normalized clonality between cell types.

Donor	Cell type	# Sorted cells	Extracted DNA (ng)	Input DNA (ng)	#TCRs	#Clono-types
1491	TCM	1,548,810	1970	417	36,178	24,606
1491	TEM	2,594,494	1994	417	33,464	13,026
1491	GPR56+ TEMRA	294,070	488	391	7,825	243
1491	GPR56- TEMRA	48,277	232	185	3,931	2,730
1554	TCM	1,019,108	568	363	11,117	10,141
1554	TEM	2,279,877	2947	417	31,602	14,173
1554	GPR56+ TEMRA	57,356	158	126	3,042	1,406
1554	GPR56- TEMRA	120,044	270	216	8,428	6,322

Supplemental Table 7A. Sample overview for targeted TCR sequencing.

	Cycles	Yield	Projected Yield	Aligned (%)	Error Rate (%)	Intensity Cycle 1	%≥Q30
**Read 1**	156	3.71 Gbp	3.71 Gbp	6.45	0.48	221	96.27
**Read 2 (I)**	15	334.96 Mbp	334.96 Mbp	0	0	473	96.36
**Non-Index Reads Total**	156	3.71 Gbp	3.71 Gbp	6.45	0.48	221	96.27
**Totals**	171	4.04 Gbp	4.04 Gbp	6.45	0.48	347	96.27

l have less than 1 nt error per 1000 nt sequenced
ould be between 80-100%
97.2% is best we got
vere aligned PhiX reads (expected 5%)

Lane	Read	Tiles	Density (K / mm2)	Cluster PF (%)	Phas/Prep has (%)	Reads	Reads PF	%≥Q30	Yield	Cycles Err Rated	Aligned (%)	Error Rate (%)	Error Rate 35 Cycles (%)	Error Rate 75 Cycles (%)	Error Rate 100 Cycles (%)	Intensity Cycle 1
1	1	38	996 ±20	97.29 ±0.27	0.419 / 0.302	24,590,732	23,925,496	96.27	3.71 Gbp	155	6.45 ±0.07	0.48 ±0.07	0.12 ±0.01	0.18 ±0.02	0.25 ±0.09	221 ±29
	2 (I)	38	996 ±20	97.29 ±0.27	0.000 / 0.000	24,590,732	23,925,496	96.36	334.96 Mbp	0	0.00 ±0.00	0.00 ±0.00	0.00 ±0.00	0.00 ±0.00	0.00 ±0.00	473 ±68
24.5 million reads																

Supplemental Table 7B. Summary of TCR sequencing runs.
